# An updated review of the economic burden of multiple sclerosis: direct and indirect costs

**DOI:** 10.3389/fimmu.2026.1678055

**Published:** 2026-03-03

**Authors:** Paloma Lanza-Leon, David Cantarero-Prieto, Daniel Coca de Pablo, Ana Colon Lopez De Dicastillo, Lianna Tarquini Rodriguez, Javier Riancho

**Affiliations:** 1 Departamento de Economía, Universidad de Cantabria, Santander, Spain; 2 Health Economics Research Group, Valdecilla Biomedical Institute Research - IDIVAL, Santander, Spain; 3 Santander Financial Institute – SANFI, Santander, Spain; 4 Department of Pharmacy, Hospital General Sierrallana, Torrelavega, Spain; 5 Department of Neurology, Hospital General Sierrallana, Torrelavega, Spain; 6 Department of Medicine and Psychiatry, University of Cantabria, Santander, Spain; 7 Neurodegenerative diseases Research Group, Valdecilla Biomedical Institute Research - IDIVAL, Santander, Spain; 8 CIBERNED, Madrid, Spain

**Keywords:** cost, disability, disease modifying therapies, multiple sclerosis, systematic review

## Abstract

Multiple sclerosis (MS) is a chronic, inflammatory, autoimmune disease of the central nervous system and represents one of the most common causes of accumulated disability in young adults. Although, currently in the vast majority of cases MS is not a determinant of death, its effects on patients can result in considerable health problems. This research provides new estimations of the total economic burden (direct -based on the difference in the total average annual amount vs matched controls without MS - and indirect costs- e.g., labor market productivity losses (premature death, presenteeism, and absenteeism losses, costs of paid and unpaid caregivers, home changes-). A literature review of English language studies published in the last 6 years is conducted to analyze the costs of MS. We search PubMed, Web of Science and Scopus as databases. The search identified 131 unique records, 31 of which met the inclusion criteria. Living with MS is expensive as a significant chronic disease. The important cost determinants are the direct costs of drugs and indirect productivity loss. Our findings show that the burden of MS needs to overcome the underestimation problems.

## 1 Introduction

Multiple sclerosis (MS) is the most common chronic inflammatory disease of the central nervous system (CNS), affecting around 2 million people worldwide (1, 2), with its prevalence continuing to rise globally (3). This demyelinating disease usually presents in early to mid-adulthood although the age of onset seems to be increasing during last year’s (4). Most people with MS (pwMS) exhibit relapsing-remitting MS forms (RRMS) characterized by fully or partially reversible episodes of neurological disability, which commonly last from days to a few weeks. As disease progresses a substantial proportion of patients with relapsing-remitting multiple sclerosis (RR-MS) transition to a progressive clinical course, which is typically associated with greater disability and a significant decline in quality of life (1). In this regard, 25 years after onset, >80% of patients had developed secondary progressive forms (5). Although MS does not have curative treatment, in recent decades, and particularly in the last 10 years, various disease-modifying treatments (DMTs) have emerged and are now contributing to changing the natural history of the disease. Intriguingly, the median duration to conversion to progressive forms increased up to 32.4 years (95% confidence interval (95% CI): 31.1-33.7) when effectively treated with MS disease-modifying therapies (6). Typically, treatments for MS are divided into moderately effective treatments (platform drugs, teriflunomide, and fumarates), highly effective drugs (cladribine and sphingosine-1-phosphate [S1P] receptor modulators, anti-CD20 drugs, natalizumab, and alemtuzumab) (7). Additionally, it is often necessary to use symptomatic treatments to ameliorate the residual symptoms of patients (such as gait disorders, affective disorders, neuralgiform pain, or genitourinary symptoms, among others) (7). Patients with MS, regardless of the stage of the disease they are in, require periodic clinical follow-up as well as various complementary studies, including blood tests, neurophysiological studies, and periodic neuroimaging studies (8).

The lifetime burden of MS should be considered at different levels including patients, their families and caregivers and society (9). Both direct and indirect medical costs are high because working lives of patients are often affected by symptoms, relapses and/or disability (10), and these costs increase over time as patients accrue disability (11).

The aim of this literature review was to analyze the existing literature on the economic burden of MS and to compare the different types of costs identified in the studies. Reporting these results together will help to identify trends and differences in healthcare costs between countries. This is essential for developing more effective and equitable public health strategies, since new DMTs have shifted a portion of healthcare spending from inpatient to outpatient care, while substantial indirect costs—such as loss of productivity—remain a critical concern (10). By examining and comparing these cost patterns, policymakers and other stakeholders will be able to work toward improving treatment access, resource allocation, and overall quality of life for individuals living with MS (10).

## 2 Methods

The methodology used in this article implied a literature review, which was performed in three different electronic databases (PubMed, Web of Science and Scopus), according to Preferred Reporting Items for Systematic Reviews and Meta-Analyses (PRISMA) guidelines (12). As it was mentioned before, this article aimed to analyze the most recent and relevant literature associated to the economic burden of MS, covering literature for the last 6 years (from 2019 to 2024). In particular, the literature search was performed on December 29, 2024.

Four key concepts were combined during the search in each database: Multiple Sclerosis AND economic burden OR direct cost OR indirect cost. Eligibility criteria were initially designed to select for original articles analyzing the costs of MS, being published in English, in peer-reviewed and open access journals. Thus, other types of documents such as reviews, comments or editorials; articles that were not published in English or that did not fit our objective were excluded. Among others, reviewed variables included: i) the country where the study was carried out, ii) the currency, iii) the type of cost, and iv) the perspective. The search strategy was developed and carried out by the authors acting as reviewers (PLL and DCdP) supported by clinicians with expertise in MS (JR and AC) for those cases of doubt or disagreement.

### 2.1 Cost analysis

The cost analysis usually included direct healthcare costs, direct non-healthcare costs, and indirect costs. All data was converted to 2024 euros (€2024). Throughout the document, costs are expressed in €2024 to ensure a uniform format and comparable information.

## 3 Results

### 3.1 Identification and description of studies

**Figure 1:**
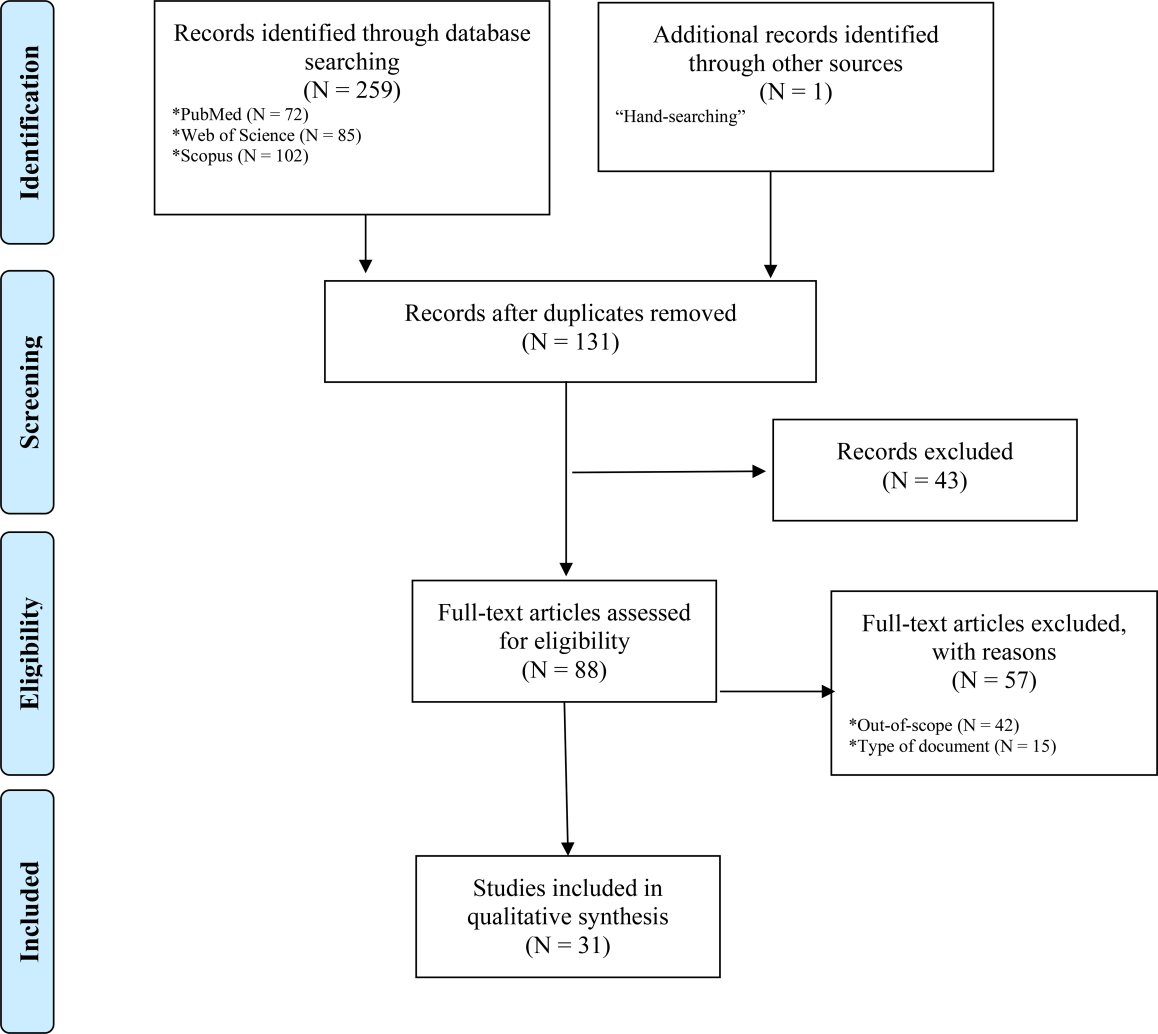
Flow diagram of the paper selection process.

A total of 259 articles were initially identified through the three databases used. In addition, one more article was identified through hand-searching. Then, once duplicated articled had been removed, the titles, abstracts and keywords of 131 articles were reviewed independently by two authors (PLL and DCdP). Of these, 43 were excluded and 88 articles were screened in full-text form to assess study eligibility based on the inclusion and exclusion criteria. Finally, 56 articles were excluded: 42 were outside our objective and 15 were not articles per se, but another type of document not containing original research such as editorial or letters. At this stage, the two authors acting as independent reviewers extracted the most important data from the 31 records identified as potentially relevant in the literature review. It should be noted that any disagreements during the study was resolved by discussion or by a third/fourth reviewer (JR and AC). Results for each stage of this screening process could be shown in **Figure 1**.

### 3.2 Included studies characteristics

The finally selected 31 studies for which cost data were available were categorized according to four variables: the country in which the research was conducted, the currency and its reference year, the type of cost evaluated (direct healthcare, direct non-healthcare, and indirect), and the analytical perspective adopted (societal, patient, or payer). Studies most often collected data from patient populations in Italy (N = 6), followed by Australia (N = 3), France (N = 3), Germany (N = 3), Canada (N = 2), China (N = 2), Spain (N = 2), Sweden (N = 2) and United States (N = 2). The remaining studies analyzed patients from Georgia, Hungary, Jordan, Kuwait, Lebanon, Malaysia and Slovakia, independently.

Eight studies (13–20) included data only related to direct costs of MS and, hence, these articles were carried out from the payer perspective. Another three articles considered information only associated with indirect costs, which followed the patient perspective (21–23). The remaining twenty studies involved direct and indirect costs (24, 43). The latter articles considered the social perspective.

### 3.3 Primary cost categories

Building upon the aforementioned classification, the following sections will examine these studies based on the analytical perspective employed: (i) payer, (ii) patient, and (iii) societal viewpoints.

#### 3.3.1 Payer perspective (direct costs)

This perspective focuses on healthcare system expenditures related to MS, particularly pharmacological treatments and medical services. In France, Vandhuick et al. (16) and Detournay et al. (14) reported annual direct costs of €35,597 and €15,296 per patient, respectively, mainly driven by pharmacotherapy and outpatient care. In Italy, Perrone et al. (18) observed that treated patients incurred up to four times higher costs (€14,120) than untreated ones, with drug acquisition and clinical monitoring as key drivers. Petruzzo et al. (15) found that the introduction of new DMTs, such as natalizumab, progressively raised average costs to €29,000 by the end of the study period.

Alowayesh et al. (13) reported a sharp rise in annual costs in Kuwait from 2011 to 2015, raising to €19,690.90, with DMTs accounting for nearly 90% of total expenses. Khakban et al. (19) contributed additional insight from Canada, where MS patients incurred an average annual excess cost of €6,846.42, underscoring the cumulative financial impact of long-term treatment. Rajakumar et al. (20) highlighted the cost-saving potential of biosimilars in Malaysia, with rituximab (Truxima) costing €7,853.08.

#### 3.3.2 Patient perspective (indirect costs)

This perspective highlights the socioeconomic impact of MS from the patient’s viewpoint, with a focus on productivity loss, early retirement, and caregiving. These studies confirm that MS imposes a substantial indirect burden (driven by fatigue, disability progression, and work disruption) underscoring the need for policies that support employment retention and reduce caregiver strain. In Canada, Rodriguez Llorian et al. (23) assessed 512 employed individuals with MS and found that more than half reported productivity loss over the previous three months, totaling an average of 60 hours lost per person (23 hours, 19 hours, and 18 hours due to presenteeism, absenteeism, and unpaid work, respectively). These losses were translated into a mean cost of €2,012.82 per patient. In the United States, Bonafede et al. (22) evaluated over 3,000 MS patients receiving DMTs and compared to matched-healthy controls. Their findings showed that MS patients had significantly greater costs associated with absenteeism, short-term disability, and long-term disability, raising to €9,220.68. Finally, from Spain, García-Domínguez et al. (21) analyzed data from 199 patients, the majority of whom had a low degree of physical disability. Despite this, the percentage of employed patients went down from 70% at diagnosis to 47% at the time in which the study was performed. Estimated annual indirect costs reached €13,014.08 per patient, primarily due to premature retirement and sick leave.

#### 3.3.3 Societal perspective (direct and indirect costs)

A further set of seventeen studies concurrently assessed direct healthcare costs, direct non-healthcare expenses, and indirect costs, yielding valuable insights into the broader economic impact of MS. Bouleau et al. (35) quantified the socioeconomic impact of MS in France, estimating a total (direct and indirect) MS cost of €32,435.00, with 48.1% due to productivity loss and informal caregiving and 51.9% attributable to direct costs. From Spain, Oreja-Guevara et al. (41) studied cohorts with Secondary Progressive MS, highlighting the increasing burden of hospitalizations and caregiver services in moderate to severe stages. The total annual costs amount to €48,547.00, of which €15,325.00 correspond to direct health costs, €8,701.00 are related to direct non-health costs, and €24,520.00 to indirect costs.

In China, Sun et al. (42) estimated the healthcare resource utilization and economic burden of MS patients, revealing a substantial total annual cost per patient of €179,121.90. This total cost includes both direct health and non-health expenses (€69,402.90, and €13,579.91, respectively), as well as indirect costs (€96,139.09). Notably, the study found that indirect costs increased significantly with disease severity, becoming the dominant component of the total cost in patients with higher EDSS scores. The findings underscore the economic impact of MS beyond the healthcare system, highlighting the societal burden due to reduced employment and work capacity. Meanwhile, Guojun et al. (36) also addressed the total economic burden (€8,650.00), it emphasized hospital-based direct costs (€4,204.00), such as inpatient care, diagnostic procedures, and treatments. Although indirect costs were acknowledged (€1,718.00), the study primarily quantified direct expenditures, revealing considerable variability in total costs across hospitals. This variation was attributed to differences in treatment protocols, resource availability, and regional healthcare policies.

From Lebanon, Dahham et al. (38) estimated an annual per-patient cost of approximately €32,779.82, of which 52% was tied to healthcare (€17,009.49, and €2,694.20 related to direct costs) and another 40% to productivity losses and informal care (€13,076.13), underscoring the societal toll in under-resourced settings. In Germany, Müller et al. (28) further confirmed RRMS as the costliest MS phenotype, averaging €20,441.00 annually, with direct costs rising in progressive forms, accounting for a total of €18,970.00. On the other hand, Dillon et al. (39) used NeuroTransData registry data to show that severe MS led to annual costs exceeding €33,263.00, with about half linked to lost wages and informal care (€17,781.00). Schriefer et al. (30) concluded that while the overall economic burden of RRMS is comparable between genders, the distribution of costs differs, reflecting broader gender-based disparities in healthcare access, employment, and caregiving roles. In this sense, the main driver of total societal costs (€11,913.00) was indirect costs (€9,853.00), particularly in patients with more advanced disease.

From Italy, several studies presented complementary findings. Ponzio et al. (40) focused on the economic impact of MS comorbidities and showed that more than half of the MS patients studied had at least one comorbid condition (hypertension, depression and anxiety, etc.). These comorbidities significantly increased health care utilization and associated costs, reaching €27,864.85 as total costs per year per patient. Meanwhile, Battaglia et al. (34) provided a detailed cost-of-illness analysis for patients with MS, highlighting the substantial economic burden MS imposes. The total annual cost per patient was estimated at €46,697.00, with indirect costs (€17,825.00) representing a significant share. Rosato et al. (32) explored the cost of illness in patients with severe MS and evaluated the cost-effectiveness of a palliative care intervention. The authors report an average total annual cost of more than €28,089.00 per patient, almost half of which is directly borne by patients and their families. In addition, Polistena et al. (25) evaluated the social impact of natalizumab treatment in MS patients, reporting a total annual cost per patient of €29,511.39, with a significant portion attributed to indirect costs (€11,125.00) and direct health costs €13,088.17).

In Australia, Ahmad et al. (26) provided an updated analysis of the economic burden of MS, finding that total annual costs per person with MS were €69,121.68. Also in this country, Brown et al. (31) investigated the societal costs of primary progressive MS and modeled the potential economic impact of a hypothetical DMT that could delay disease progression, achieving total costs €69,451.50, and indirect costs accounted for 67.5% of this total (€46,885.41). On the other hand, Palmer et al. (29) adopted a lifetime perspective, modeling total lifetime societal costs for a hypothetical cohort of patients. The results show that the total cost per patient amounted to €50,643.66, disaggregated as €7,850.99 for direct health costs and €32,792.18 for indirect costs.

From Sweden, Karampampa et al. (27) identified four distinct cost trajectories and showed that, while direct healthcare costs peak shortly after diagnosis and then decline, indirect costs tend to increase over time. In particular, the total cost involved €29,278.00 per patient and year. Gyllensten et al. (24) investigated how disease costs evolve in the different MS phenotypes, concluding that total costs increase significantly with disease progression and reach €24,760.33 per patient per year.

Finally, Alabbadi et al. (43) conducted one of the first comprehensive analyses of the cost of MS disease in Jordan, revealing a total annual cost per patient of €11,598.98, of which the largest share (€11,136.75) is for direct healthcare costs and a smaller share (€462.22) for indirect costs. Similarly, in Slovakia, Babela and Dugas (33) presented a retrospective analysis of the costs associated with MS, providing one of the first systematic attempts to quantify the financial impact of the disease. They suggested that the total economic burden of MS per patient per year was €7,978.00.

**Table 1** summarizes and compares the cost components reported in the 31 studies included in our analysis. The table presents data on direct health costs, direct non-health costs, and indirect costs, as well as total costs, across various countries. To facilitate cross-country comparisons, all values have been converted to 2024 euros. This harmonized overview provides insight into the variability of economic burden estimates depending on methodological choices, cost components included, and national contexts.

**Table 1:** Mapping and comparing costs (€2024) of the 31 studies included in the analysis.

Article	Country	Direct health costs	Direct non-health costs	Indirect costs	Total costs
Alabbadi et al. (43)	Jordan	€11,136.75	N/I	€462.22	€11,598.98
Oreja-Guevara et al. (41)	Spain	€15,325.00	€8,701.00	€24,520.00	€48,547.00
Rajakumar et al. (20)	Malaysia	€7,853.08	N/I	N/I	N/I
Sun et al. (42)	China	€69,402.90	€13,579.91	€96,139.09	€179,121.90
Dahham et al. (38)	Lebanon	€17,009.49	€2,694.20	€13,076.13	€32,779.82
Dillon et al. (39)	Germany	€11,228.000	€4,254.00	€17,781.00	€33,263.00
Khakban et al. (19)	Canada	€6,846.42	N/I	N/I	N/I
Ponzio et al. (40)	Italy	€15,260.39	€3,998.35	€8,606.11	€27,864.85
Babela and Dugas (33)	Slovakia	€7,422.00	N/I	€556.00	€7,978.00
Battaglia et al. (34)	Italy	€25,030.00	€3,842.00	€17,825.00	€46,697.00
Bouleau et al. (35)	France	€16,881.00	N/I	€15,554.00	€32,435.00
Gugutsidze et al. (17)	Georgia	€7,382.50	N/I	N/I	N/I
Guojun et al. (36)	China	€4,204.00	N/I	€1,718.00	€8,650.00
Le et al. (37)	United States	€16,262.94	N/I	€19,846.51	€36,109.45
Perrone et al. (18)	Italy	€14,120.00	N/I	N/I	N/I
Rodriguez Llorian et al. (23)	Canada	N/I	N/I	€2,012.82	N/I
Bonafede et al. (22)	United States	N/I	N/I	€9,220.68	N/I
Brown et al. (31)	Australia	€22,566.08	N/I	€46,885.41	€69,451.50
Rosato et al. (32)	Italy	€15,874.00	N/I	€12,215.00	€28,089.00
Vandhuick et al. (16)	France	€35,597.00	N/I	N/I	N/I
Ahmad et al. (26)	Australia	€30,674.27	€6,411.62	€32,035.79	€69,121.68
Karampampa et al. (27)	Sweden	€10,392.00	N/I	€18,887.00	€29,278.00
Müller et al. (28)	Germany	€18,970.00	N/I	€1,472.00	€20,441.00
Palmer et al. (29)	Australia	€7,850.99	N/I	€32,792.18	€50,643.66
Petruzzo et al. (15)	Italy	€29,000.00	N/I	N/I	N/I
Schriefer et al. (30)	Germany	€1,956.00	€224.00	€9,853.00	€11,913.00
Alowayesh et al. (13)	Kuwait	€19,690.90	N/I	N/I	N/I
Detournay et al. (14)	France	€15,296.00	N/I	N/I	N/I
García-Domínguez et al. (21)	Spain	N/I	N/I	€13,014.08	N/I
Gyllensten et al. (24)	Sweden	€12,149.97	N/I	€12,610.36	€24,760.33
Polistena et al. (25)	Italy	€13,088.17	€5,298.22	€11,125.00	€29,511.39

N/I (Not included). Source: Authors’ elaboration.

### 3.4 Limitations

This review has some limitations that should be considered. First, the inclusion criteria were not homogeneous across studies, with some of them including specific MS forms and others assessing all types of patients. Because of to the reduced number of studies included for the review, we decided to perform a global discussion of the literature. Second, many articles had missing data, which led to their exclusion from the review. Thirdly, within the analysis of direct treatment-related costs, the availability of therapies—particularly those classified as high- and very high-efficacy—was not consistent across all included studies. This heterogeneity may influence the results and introduce cross-country bias. Finally, very few studies reported on direct non-health costs related to MS. That is, this study presents a narrative literature review on the costs of MS. The included articles assess diverse cost components, often employing different methodologies and variables. As a result, the reported cost estimates may vary across studies due to inconsistencies in data collection, cost categories, and analytical frameworks.

## 4 Conclusion

This review presented a comprehensive and recent overview of the costs of MS. The different articles considered highlighted the high economic burden of this disease for both the health care system and the patient and family members. Direct non-health costs are the least quantified in the studies. For this reason, further analysis in these areas is required.
